# Unexpected chiral vicinal tetrasubstituted diamines via borylcopper-mediated homocoupling of isatin imines

**DOI:** 10.3762/bjoc.18.34

**Published:** 2022-03-10

**Authors:** Marco Manenti, Leonardo Lo Presti, Giorgio Molteni, Alessandra Silvani

**Affiliations:** 1 Dipartimento di Chimica, Università degli Studi di Milano, Via Golgi 19, Milano, 20133, Italy

**Keywords:** atropoisomer, bis(pinacolato)diboron, 3,3′-bisoxindole, *N*-*tert*-butanesulfinyl ketimine, homocoupling

## Abstract

Addressing the asymmetric synthesis of oxindole-based α-aminoboronic acids, instead of the expected products we disclosed the efficient homocoupling of oxindole-based *N*-*tert*-butanesulfinyl imines, with the generation of chiral, quaternary 1,2-diamines in a mild and completely stereoselective way. The obtained 3,3′-bisoxindole derivatives were fully characterized by NMR and single-crystal X-ray diffraction analysis and proved to be single diastereoisomers and atropisomers. A plausible mechanism for the one-pot Cu(II)-catalyzed Bpin addition to the isatin-derived ketimine substrate and subsequent homocoupling is described.

## Introduction

As bioisosteres of carboxylic acid derivatives, boronic acids have recently emerged as a novel chemotype in drug design, with a number of boron-containing compounds recently being approved by the FDA [1–4]. In particular, α- and β-aminoboronic acids are commonly utilized as key intermediates for the synthesis of boron-containing peptidomimetics, which have been demonstrated to be efficient covalent ligands and valuable protease inhibitors endowed with various biological activities [5–6].

Going on with our interest in the synthesis of 3,3-disubstituted oxindoles [7–11] and also of aminoboronic acids [12], we recently exploited a molecular hybridization strategy to synthesize chiral oxindole-based β-aminoboronic acids and spiro derivatives [13]. Apart from our work and a quite recent report describing a useful Cu-catalyzed enantioselective intramolecular transformation [14], the insertion of a boron atom into chiral oxindoles is scarcely reported.

Continuing with such previous project, next we looked at the copper-mediated reaction of isatin-derived, optically pure sulfinyl ketimines with bis(pinacolato)diboron, as a potential way to access oxindole-based α-aminoboronates. The asymmetric synthesis of diverse α-aminoboronic acids by diastereoselective Cu(I)-catalyzed borylation of *N*-*tert*-butanesulfinyl aldimines was described by Ellman and co-workers for the first time in 2008 [15] and next further developed with a more stable Cu(II) catalyst in 2014 [16].

Herein, we describe the unexpected results achieved by our work, that is the obtainment of unprecedented, bisoxindole-based, vicinal, tetrasubstituted diamines. Bisoxindoles represent a particularly intriguing class of compounds, because of their interesting biological activities and because they can serve as key synthetic intermediates in the construction of complex natural products [17–23]. Particularly challenging is the placement of the two C3/C3’ contiguous quaternary stereogenic centers, just as they are found in various alkaloids, such as those belonging to the bis(cyclotryptamine) family [24–27].

In light of these considerations and as, to our knowledge, no borylcopper-mediated homocoupling of *N*-*tert*-butanesulfinyl imines have been documented before, we consider useful to share our findings and to accurately describe the obtained products.

## Results and Discussion

**Scheme 1:**
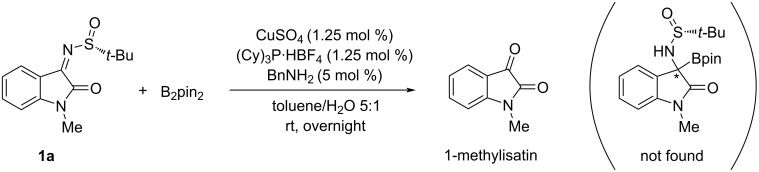
Reaction conducted according to the Ellman protocol.

We began our investigation using the known (*R*)-1-methylisatin-derived *N*-*tert*-butanesulfinyl ketimine **1a**, bis(pinacolato)diboron, CuSO_4_/(Cy)_3_P catalyst and benzylamine, as reported in Scheme 1. The first reaction was carried out at room temperature in toluene/water (5:1), as described by Ellman and co-workers, but the expected α-aminoboronate could not be isolated. Extensive hydrolysis of the starting ketimine occurred, allowing only the recovery of the 1-methyl-isatin precursor.

In the light of this initial outcome, we started a detailed screening of reaction conditions (Table 1), evaluating firstly different amines (Table 1, entries 1–5), in place of benzylamine. Using DIPEA, the new product **2a** was obtained in 31% yield, even if together with 1-methylisatin and other impurities. Aiming to improve the fruitful conversion of substrate **1a**, we considered to reduce the amount of water to the minimum necessary to solubilize the copper sulfate. Thus, performing the reaction in toluene/water 100:1, ketimine hydrolysis was almost entirely prevented and the yield of compound **2a** raised to 52% (Table 1, entry 6). With regard to the ligand (Table 1, entries 7 and 8), PPh_3_ behaved most effectively, further promoting the conversion of the substrate. Other changes in reaction conditions, such as heating at 70 °C, increasing the amount of copper catalyst to 10 mol % and switching the copper salt from CuSO_4_ to the more soluble Cu(OTf)_2_, did not improve the yield significantly (Table 1, entries 9–11), while the yield raised up to 68% when 0.5 equivalents of base were used (Table 1, entry 12). Finally, in order to shed light on the reaction mechanism (see below), three control experiments were carried out. Under anhydrous conditions (Table 1, entry 13), conversion of the starting ketimine did not take place. Also conducting the reaction in the absence of B_2_pin_2_ (Table 1, entry 14) or copper salt (Table 1, entry 15), respectively, no reaction could be observed and the substrate was recovered together with small amounts of the 1-methylisatin precursor.

**Table 1:** Screening of the reaction conditions for compound **2a**.^a^

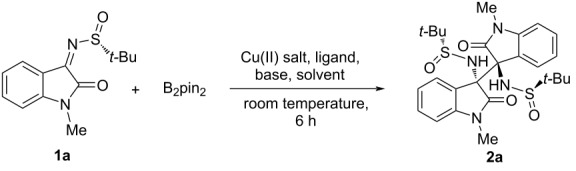					

entry	Cu(II) salt	ligand	base	solvent	yield **2a** [%]^b^

1	CuSO_4_	(Cy)_3_P·HBF_4_	Py	toluene/H_2_O 5:1	6
2	CuSO_4_	(Cy)_3_P·HBF_4_	DMAP	toluene/H_2_O 5:1	n. d.
3	CuSO_4_	(Cy)_3_P·HBF_4_	DABCO	toluene/H_2_O 5:1	n. d.
4	CuSO_4_	(Cy)_3_P·HBF_4_	TEA	toluene/H_2_O 5:1	23
5	CuSO_4_	(Cy)_3_P·HBF_4_	DIPEA	toluene/H_2_O 5:1	31
6	CuSO_4_	(Cy)_3_P·HBF_4_	DIPEA	toluene/H_2_O 100:1	52
7	CuSO_4_	(o-tol)_3_P	DIPEA	toluene/H_2_O 100:1	62
8	CuSO_4_	(Ph)_3_P	DIPEA	toluene/H_2_O 100:1	66
9^c^	CuSO_4_	(Ph)_3_P	DIPEA	toluene/H_2_O 100:1	33
10^d^	CuSO_4_	(Ph)_3_P	DIPEA	toluene/H_2_O 100:1	61
11	Cu(OTf)_2_	(Ph)_3_P	DIPEA	toluene/H2O 100:1	66
12^e^	CuSO_4_	(Ph)_3_P	DIPEA	toluene/H_2_O 100:1	68
13	CuSO_4_	(Ph)_3_P	DIPEA	toluene/DMSO 100:1	n. r.
14^f^	CuSO_4_	(Ph)_3_P	DIPEA	toluene/H_2_O 100:1	n. r.
15^g^	–	–	DIPEA	toluene/H_2_O 100:1	n. r.

^a^Reagents and conditions: ketimine **1a** (0.2 mmol), B_2_pin_2_ (1.5 equiv), Cu(II) salt (2.5 mol %), ligand (2.5 mol %), base (5 mol %), in solvent (0.7 M). ^b^Isolated yields. ^c^Reaction heated at 70 °C. ^d^Reaction performed with 10% of CuSO_4_ and PPh_3_. ^e^Reaction performed with 0.5 equiv of DIPEA. ^f^Reaction performed in the absence of B_2_pin_2_. ^g^Reaction performed in the absence of Cu(II) salt and ligand. n. d. = not determined. n. r. = no reaction.

From the reaction run under the optimized conditions (Table 1, entry 12) followed by flash chromatography, the unprecedented bisoxindole **2a** was fully characterized by high-resolution mass spectrometry and by one- and two-dimensional NMR analysis. In particular, from HSQC, HMBC and COSY experiments all single frequencies could be safely assigned in the ^1^H and ^13^C NMR spectra, allowing the complete spin system reconstruction for both the oxindole-based units. Furthermore, from NOESY experiment, the unique *anti* disposition of the two *t-*Bu–SO–NH substituents along the C3–C3’ bond could be assessed, thus demonstrating the complete diastereoselectivity of the reaction (see Supporting Information File 1).

**Figure 1:**
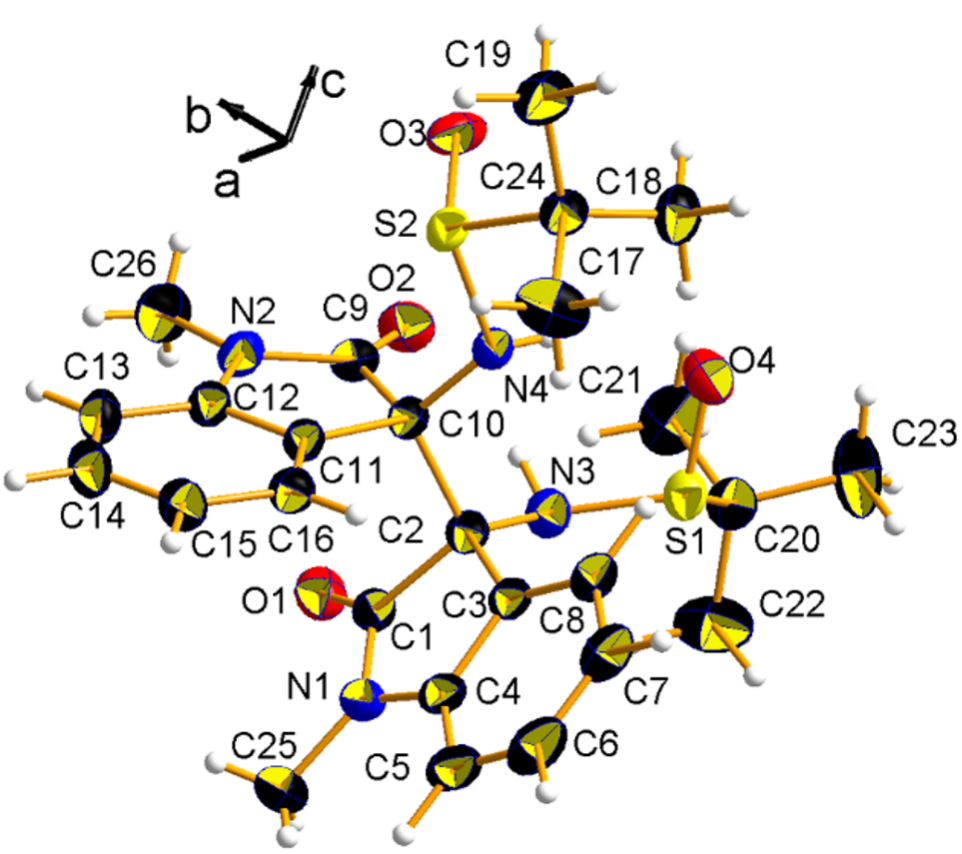
Asymmetric unit of **2a**, with the atom-numbering scheme. The crystallographic reference system is also shown. Thermal ellipsoids at rt were drawn at the 50% probability level. Atoms are represented with the usual colour code (C: black; N: blue; O: red; S: yellow; H: white).

All stereochemical implications were fully confirmed by single-crystal X-ray diffraction analysis, which was performed on well-formed prismatic crystals of compound **2a** (Figure 1) [28].

Compound **2a** is chiral and crystallizes in the orthorhombic space group *P*2_1_2_1_2_1_. The presence of sulfur anomalous scatters allowed to unequivocally establish the absolute configurations, which reads *S* at the chiral center C2 and *R* at the C10 (C numbering as in Figure 1). The absolute configuration of the two sulfur stereogenic centers is confirmed to be *R*. Interestingly, the molecule does not bear any pseudo-mirror plane, that is, the observed conformer is asymmetric (C1) in itself, regardless the configuration of the two sulfinamide substituents. This peculiarity is likely due to the hindered rotation across the newly formed C–C bond, joining the two oxindole units. Such the C2–C10 single bond is quite long (1.58 Å), as expected due to crowding of the two facing oxindole systems. In the crystal, NH groups set up intramolecular hydrogen bonds with the O acceptors of the sulfinamide moieties (see Table 2), likely contributing to further stabilize the observed conformer.

**Table 2:** Intramolecular hydrogen bonds in **2a** at room temperature, which involve the NH groups with one keto oxygen (O2) and one sulfinamide oxygen (O4). Atom numbering as in Figure 1. The asymmetry of such interactions reflects the intrinsic asymmetry of the solid-state conformer.

D–H···A	*d*_D-H_ [Å]	*d*_H…A_ [Å]	*d*_D-A_ [Å]	α [deg]	symmetry

N3–H3N···O2	0.99(4)	2.03(4)	2.854(4)	139(3)	x, y, z
N4–H4N···O4	0.82(4)	2.21(4)	2.981(4)	157(4)	x, y, z

**Figure 2:**
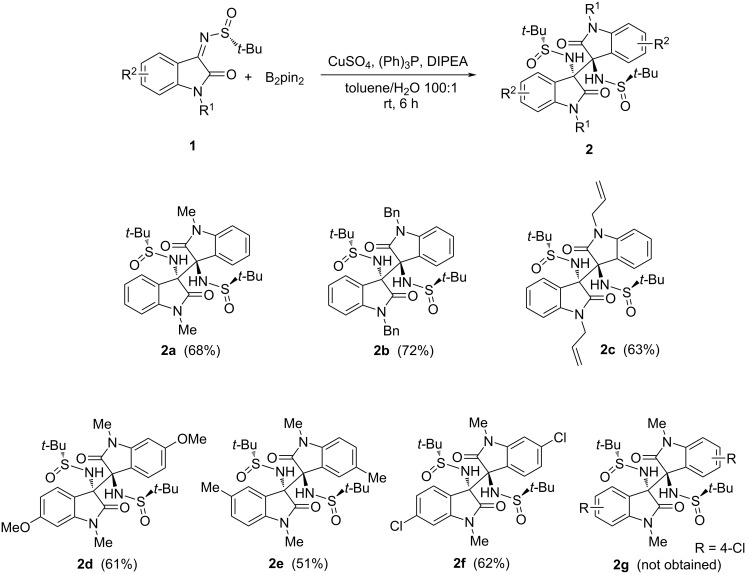
Substrate scope of the borylcopper-mediated homocoupling of oxindole-based *N*-*tert*-butanesulfinyl imines **1** (isolated yields in parentheses).

Aiming to generalize the discovered transformation, a brief scope of the reaction with respect to the *N*-*tert*-butanesulfinyl imine substrate was next performed (Figure 2). The protecting group R^1^ on the oxindole nitrogen atom was found to have a moderate effect on the reactivity, with R^1^ = Bn giving the best yield (**2a–c**). *N*-Methylisatin ketimines with various R^2^ residues were also evaluated. In the presence of substituents at the C5 or C6 position on the oxindole aromatic ring, such as electron-donating groups (6-OMe, **2d** and 5-Me, **2e**) and halogen substituents (6-Cl, **2f**), good yields of the corresponding quaternary 1,2-diamines were obtained. Instead, the presence of a substituent at the C4 position (4-Cl, **2g**) hinders the course of the reaction, likely due to its spatial proximity with the reaction center, and no homocoupling could be observed.

**Scheme 2:**
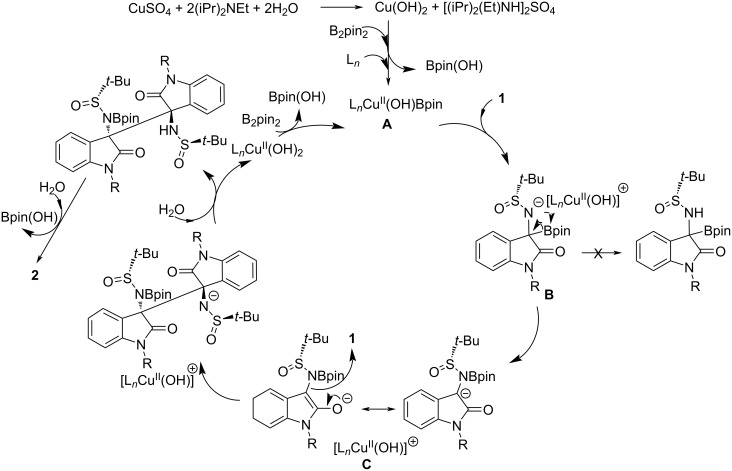
Proposed mechanism for the borylcopper-mediated homocoupling of ketimines **1**.

In order to rationalize the formation of bisoxindole products, besides relying on performed control experiments, we also refer to the underdeveloped umpolung reactions of imines, considering, in particular, the copper-catalyzed process reported quite recently by Zhang, Hou and co-workers [29]. In our case, we presume the possible reaction mechanism shown in Scheme 2, which likely starts from the catalytic generation, in our experimental conditions, of the borylcopper(II) species **A**. Such copper complex proved to be not isolable, but could be easily generated in situ and may act as a genuine Cu(II) catalyst, with a labile coordination site [30]. According to Ellman’s chemistry, the addition of Bpin to the C=N double bond of the ketimine substrate **1** should actually take place, affording the intermediate **B**, the immediate precursor of our original target compound, namely the α-aminoboronate derivative. However, probably due to its high steric crowding, such intermediate spontaneously turns into the carbanion **C**, thus realizing the imine umpolung and allowing the cross-coupling reaction with the remaining electrophilic ketimine **1**. The complete diastereoselectivity would arise from the mutual approach of the two oxindole nuclei from the less hindered side, that is the one away from the bulky auxiliary *t-*Bu group. The presence of two NH-SO*t-*Bu substituents, preventing the free rotation around the C3–C3’ bond, ensures the optical activity of the molecule, in accordance with the presence of a single atropoisomer (absence of any pseudo-mirror plane), as also determined by single-crystal X-ray diffraction analysis.

## Conclusion

In summary, we have disclosed a reaction protocol that allows efficient homocoupling of oxindole-based *N*-*tert*-butanesulfinyl imines and generation of chiral, quaternary 1,2-diamines in a mild and completely stereoselective way. The one-pot, simple experimental procedure makes this process a convenient and straightforward approach for the synthesis of enantiomerically pure vicinal diamines and structurally challenging bisoxindole natural products.

## Supporting Information

File 1 Experimental part, NMR spectra and christallographic data of compound **2a**.
