# Supplementary material for: Unexpected chiral vicinal tetrasubstituted diamines via borylcopper-mediated homocoupling of isatin imines
Source: Beilstein J Org Chem. 2022 Mar 10;18:303–8. doi: 10.3762/bjoc.18.34 (PMC8919417; doi:10.3762/bjoc.18.34)
Supplement: File 1 — Experimental part, NMR spectra and christallographic data of compound 2a. [file Beilstein_J_Org_Chem-18-303-s001.pdf]

BEILSTEIN JOURNAL OF ORGANIC CHEMISTRY

Supporting Information

for

Unexpected chiral vicinal tetrasubstituted diamines via borylcopper-mediated homocoupling of isatin imines

Marco Manenti, Leonardo Lo Presti, Giorgio Molteni and Alessandra Silvani

Beilstein J. Org. Chem. **2022,** *18,* 303–308. doi:10.3762/bjoc.18.34

Experimental part, NMR spectra and christallographic data of compound 2a

License and Terms: This is a supporting information file under the terms of the Creative Commons Attribution License (https://creativecommons.org/licenses/by/4.0). Please note that the reuse, redistribution and reproduction in particular requires that the author(s) and source are credited and that individual graphics may be subject to special legal provisions.
The license is subject to the *Beilstein Journal of Organic Chemistry* terms and conditions: (https://www.beilstein-journals.org/bjoc/terms)

Table of contents

General Information	S2
General Procedure for the synthesis of compounds **2**	S2
^{1}H and ^{13}C NMR spectra analysis for compound **2a**	S6
Copies of ^{1}H and ^{13}C NMR spectra	S8
Crystallographic data for compound **2a**	S14
References	S16

General Information

^{1}H NMR and ^{13}C NMR spectra were recorded using a Bruker AV 400 Ultrashield spectrometer. ^{1}H NMR and ^{13}C NMR chemical shifts were reported in parts per million (ppm) downfield from tetramethylsilane. Coupling constants (*J*) were reported in Hertz (Hz). The residual solvent peaks were used as internal reference: ^{1}H NMR (CD_3CN 1.98 ppm) ^{13}C NMR (CD_3CN 0.3 ppm, 117.3 ppm). The following abbreviations were used to explain the multiplicities: s = singlet, d = doublet, t = triplet, m = multiplet, br = broad. All the *N*-substituted isatins[1] and the corresponding ketimines were synthetized according to the previous literature and their spectroscopic data are in agreement with the reported ones.[2,3] Other reagents were received from commercial sources (Fluorochem, TCI and Merck) and used without further purifications. Column chromatography were performed by flash chromatography (FC) using Merck Silica gel 60. When the reaction was scaled up, traces of pinacol persists after FC and can be seen in NMR spectra (^{1}H NMR 400 MHz, CD_3CN δ 4.16 br s, 1.15 s; ^{13}C NMR 101 MHz, CD_3CN δ 22.1, quaternary carbons not observed).

General procedure for the synthesis of compounds 2

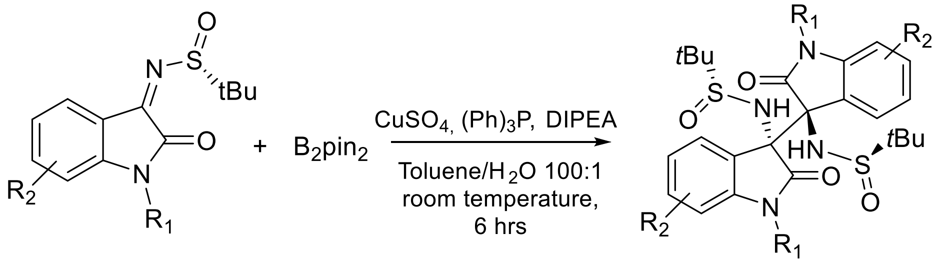

1 **2**

In a round-bottom flask, $(Ph)_3P$ (0.025 equiv) was dissolved in dry toluene (0.024 M). Saturated aqueous solution of copper sulfate pentahydrate (0.025 equiv 0.78 M) was added followed by DIPEA (0.5 equiv) and the biphasic mixture stirred at high speed (1500 rpm) for 10 minutes (aqueous phase goes from a dark-blue to a pale blue colour). B_2pin_2 (1.5 equiv) was added followed by a solution of **1** in dry toluene (1.0 equiv 0.47 M) and the reaction stirred at high speed and room temperature until the disappearance of **1** (monitored by TLC, approx. 6 h in which the reaction goes from red/orange to brown). The reaction was diluted with dichloromethane and filtered over a short pad of celite, then the solvents were removed under reduced pressure. The mixture was diluted with dichloromethane, washed with brine, dried over anhydrous $NaSO_4$ and the solvent removed under reduced pressure. The crude mixture was purified by FC to afford pure **2**.

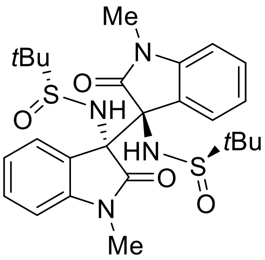

2a

*(R,R)-N,N'-((3R,3'S)-1,1'-dimethyl-2,2'-dioxo-[3,3'-biindoline]-3,3'-diyl)bis(2-methylpropane-2-sulfinamide) (**2a**)*

Synthetized according to the general procedure starting from *N*-Me-isatin-derived ketimine **1a**; purified by FC (ethyl acetate/dichloromethane 1:1) to afford a salmon pink solid (yield 68%); $[\alpha]_D$ = -136.1 (C= 1.0 in $CHCl_3$), melting point: 152-156°C, ^1H NMR (400 MHz, CD_3CN) δ 8.11 (d, *J*= 7.6 Hz, 1H), 7.58 (t, *J*= 7.6 Hz, 1H), 7.35-7.25 (m, 2H), 6.96-6.89 (m, 2H), 6.88 (s, 1H), 6.68 (t, *J*= 7.6 Hz, 1H), 6.16 (s, 1H), 5.69 (d, *J*= 7.6 Hz, 1H), 3.18 (s, 3H), 2.69 (s, 3H), 1.26 (s, 9H), 1.21 (s, 9H); ^{13}C NMR (101 MHz, CD_3CN) δ 176.7 (1C), 172.9 (1C), 145.8 (2C), 132.1 (1C), 131.6 (1C), 130.3 (1C), 126.2 (1C), 122.63 (1C), 122.57 (1C), 121.8 (2C), 109.7 (1C), 109.5 (1C), 70.0 (1C), 63.8 (1C), 57.1 (1C), 56.7 (1C), 26.7 (1C), 26.0 (1C), 22.79 (3C), 22.76 (3C); HRMS-ESI $[M+Na]^+$ calculated for $C_{26}H_{34}N_4O_4S_2Na^+$ 553.1919 found 553.1924.

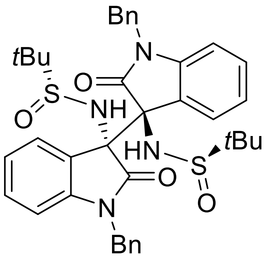

2b

*(R,R)-N,N'-((3R,3'S)-1,1'-dibenzyl-2,2'-dioxo-[3,3'-biindoline]-3,3'-diyl)bis(2-methylpropane-2-sulfinamide) (**2b**)*

Synthetized according to the general procedure starting from *N*-Bn-isatin-derived ketimine **1b**; purified by FC (dichloromethane/ethyl acetate 9:1 to 8:2) to afford a purple foam (yield 72%); $[\alpha]_D$= -126.1 (C=1.0 in $CHCl_3$); ^1H NMR (400 MHz, CD_3CN) δ 8.18 (d, *J*= 7.4 Hz, 1H), 7.63-7.55 (m, 2H), 7.45 (t, *J*= 7.4 Hz 1H), 7.36-7.22 (m, 5H), 7.20-7.07 (m, 3H), 6.99 (s, 1H), 6.77 (d, *J*= 7.6 Hz, 1H), 6.69 (d, *J*= 7.6 Hz, 1H), 6.65-6.55 (m, 3H), 6.30 (s, 1H), 5.84 (d, *J*= 7.6 Hz, 1H), 5.11 (d, *J*= 16.1 Hz, 1H), 4.81 (d, *J*= 16.1 Hz, 1H), 4.79 (d, *J*= 16.1 Hz, 1H), 4.42 (d, *J*= 16.1 Hz, 1H), 1.31 (s, 9H), 1.25 (s, 9H); ^{13}C NMR (101 MHz, CD_3CN) δ 177.3 (1C), 173.3 (1C), 145.2 (1C), 144.7 (1C), 136.5 (1C), 135.9 (1C), 131.9 (1C), 131.7 (1C), 130.3 (1C), 129.4 (2C), 129.2 (2C), 128.1 (3C), 128.0 (1C), 127.2 (2C), 126.9 (1C), 123.1 (1C), 122.8 (1C), 122.1 (2C), 110.9 (1C), 110.6 (1C), 69.8 (1C), 63.7 (1C), 57.3 (1C), 56.9 (1C), 44.7 (1C), 43.8 (1C), 22.9 (3C), 22.7 (3C); HRMS-ESI $[M+Na]^+$ calculated for $C_{38}H_{42}N_4O_4S_2Na^+$ 705.2545 found 705.2540.

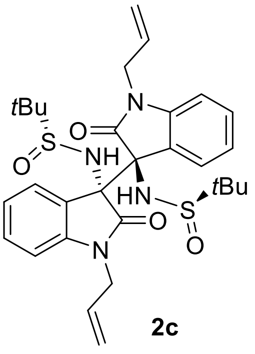

2c

*(R)-N-((3R,3'S)-1,1'-diallyl-3-(((R)-tert-butylsulfinyl)amino)-2,2'-dioxo-[3,3'-biindolin]-3'-yl)-2-methylpropane-2-sulfinamide (**2c**)*

Synthetized according to the general procedure starting from *N*-allylisatin-derived ketimine **1c**; purified by FC (Ethyl acetate / Dichloromethane 1:1) to afford a dark orange foam (yield 63%);

[α]$_D$= -143.3 (C=1.0 in $CHCl_3$); ^{1}H NMR (400 MHz, $CDCl_3$) δ 8.15 (d, *J*=7.8 Hz, 1H), 7.44 (t, *J*=7.8 Hz, 1H), 7.19 (t, *J*=7.5 Hz, 2H), 7.05 (br s, 1H), 6.79 (d, *J*=7.5 Hz, 1H), 6.73 (d, *J*=7.8 Hz, 1H), 6.61 (t, *J*=7.5 Hz, 1H), 6.04 (br s, 1H), 5.96-5.84 (m, 1H), 5.71 (d, *J*=7.5 Hz, 1H), 5.40 (app d, *J*=17.3 Hz, 1H), 5.22 (app d, *J*=10.3 Hz, 1H), 5.07-4.94 (m, 1H), 4.85 (app d, *J*=10.8 Hz, 1H), 4.64 (app. d, *J*=17.3 Hz, 1H), 4.42-4.31 (m, 2H), 4.06 (app d, *J*=15.7 Hz, 1H), 3.74 (dd, J_2=15.7 Hz, J_3=5.3 Hz, 1H), 1.32 (s, 9H), 1.24 (s, 9H); ^{13}C NMR (101 MHz, $CDCl_3$) δ 176.1 (1C), 172.0 (1C), 144.6 (1C), 144.0 (1C), 131.4 (1C), 131.1 (1C), 130.7 (1C), 130.3 (1C), 129.7 (1C), 126.0 (1C), 122.09 (1C), 122.04 (1C), 121.7 (1C), 121.4 (1C), 117.9 (1C), 117.4 (1C), 110.1 (1C), 109.4 (1C), 69.4 (1C), 62.9 (1C), 56.9 (1C), 56.7 (1C), 43.0 (1C), 41.9 (1C), 23.00 (3C), 22.96 (3C); HRMS-ESI $[M+Na]^+$ calculated for $C_{30}H_{38}N_4O_6S_2Na^+$ 605.2232 found 605.2239.

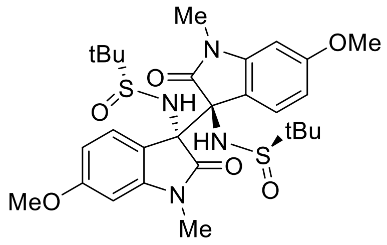

2d

*(R,R)-N,N'-((3R,3'S)-6,6'-dimethoxy-1,1'-dimethyl-2,2'-dioxo-[3,3'-biindoline]-3,3'-diyl)bis(2-methylpropane-2-sulfinamide) (**2d**)*

Synthetized according to the general procedure starting from 6-MeO-1-Me-isatin-derived ketimine **1d**; purified by FC (ethyl acetate/dichloromethane 7:3) to afford an orange foam (yield 61%); [α]$_D$= -104.5 (C=1.0 in $CHCl_3$); ^{1}H NMR (400 MHz, $CDCl_3$) δ 8.04 (d, *J*=7.5 Hz, 1H), 7.10 (s, 1H), 6.69 (d, *J*=8.3 Hz, 1H), 6.37 (s, 1H), 6.33 (s, 1H), 6.18 (d, *J*=8.3 Hz, 1H), 6.00 (s, 1H), 5.72 (d, *J*=8.3 Hz, 1H), 3.90 (s, 3H), 3.75 (s, 3H), 3,27 (s, 3H), 2.76 (s, 3H), 1.33 (s, 9H), 1.26 (s, 9H); ^{13}C NMR (101 MHz, $CDCl_3$) δ 177.1 (1C), 173.1 (1C), 162.2 (1C), 162.1 (1C), 146.6 (1C), 146.4 (1C), 130.6 (1C), 126.6 (1C), 113.4 (1C), 113.1 (1C), 107.0 (1C), 105.7 (1C), 96.3 (1C), 96.1 (1C), 69.1 (1C), 62.5 (1C), 56.63 (1C), 56.59 (1C), 55.6 (1C), 55.4 (1C), 26.7 (1C), 26.0 (1C), 24.5 (3C), 23.0 (3C); HRMS-ESI $[M+Na]^+$ calculated for $C_{28}H_{38}N_4O_6S_2Na^+$ 613.2130 found 613.2137.

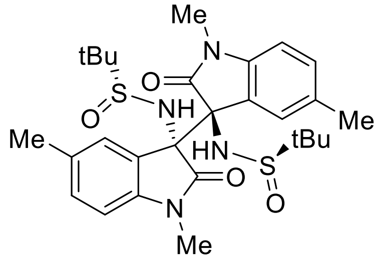

2e

*(R,R)-N,N'-((3R,3'S)-1,1',5,5'-tetramethyl-2,2'-dioxo-[3,3'-biindoline]-3,3'-diyl)bis(2-methylpropane-2-sulfinamide) (**2e**)*

Synthetized according to the general procedure starting from 5-Me-1-Me-isatin-derived ketimine **1e**; purified by FC (ethyl acetate/dichloromethane 8:2) to afford an orange foam (yield 51%); [α]$_D$= -100.1 (C=1.0 in $CHCl_3$); ^{1}H NMR (400 MHz, $CDCl_3$) δ 8.02 (br s, 1H), 7.33 (d, *J*=7.5 Hz, 1H), 7.13 (br s, 1H), 7.04 (d, *J*=7.5 Hz, 1H), 6.69 (d, *J*=7.8 Hz, 1H), 6.66 (d, *J*=7.8 Hz, 1H), 6.04 (br s, 1H), 5.35 (br

s, 1H), 3.27 (s, 3H), 2.69 (s, 3H), 2.47 (s, 3H), 1.99 (s, 3H), 1.36 (s, 9H), 1.30 (s, 9H); ^{13}C NMR (101 MHz, $CDCl_3$) δ 176.9 (1C), 172.9 (1C), 143.3 (2C), 132.3 (2C), 131.8 (1C), 131.6 (1C), 131.3 (1C), 127.1 (1C), 122.3 (1C), 122.0 (1C), 109.2 (1C), 108.5 (1C), 70.4 (1C), 63.9 (1C), 57.4 (2C), 27.3 (1C), 26.5 (1C), 23.7 (3C), 23.6 (3C), 21.9 (1C), 21.6 (1C); HRMS-ESI $[M+Na]^+$ calculated for $C_{28}H_{38}N_4O_4S_2Na^+$ 581.2232 found 581.2240.

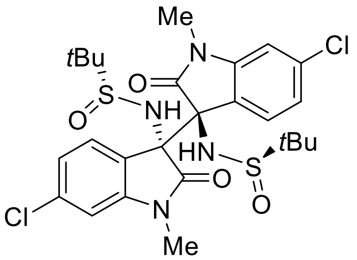

2f

*(R)-N-((3R,3'S)-3-(((R)-tert-butylsulfinyl)amino)-6,6'-dichloro-1,1'-dimethyl-2,2'-dioxo-[3,3'-biindolin]-3'-yl)-2-methylpropane-2-sulfinamide (**2f**)*

Synthetized according to the general procedure starting from 6-Cl-1-Me-isatin-derived ketimine **1f**; purified by FC (ethyl acetate/dichloromethane 1:1) to afford a dark yellow foam (yield 62%); $[\alpha]_D$= -111.7 (C=1.0 in $CHCl_3$); ^{1}H NMR (400 MHz, $CDCl_3$) δ 8.08 (d, *J*=8.3 Hz, 1H), 7.23 (d, *J*=8.3 Hz, 1H), 7.02 (br s, 1H), 6.85 (br s, 1H), 6.82 (br s, 1H), 6.71 (d, *J*=7.9 Hz, 1H), 6.11 (br s, 1H), 5.76 (d, *J*=7.9 Hz, 1H), 3.29 (s, 3H), 2.81 (s, 3H), 1.34 (s, 9H), 1.29 (s, 9H); ^{13}C NMR (101 MHz, $CDCl_3$) δ 176.3 (1C), 172.2 (1C), 146.3 (1C), 146.0 (1C), 137.4 (1C), 137.1 (1C), 130.6 (1C), 126.3 (1C), 122.3 (1C), 122.1 (1C),119.8 (1C), 119.5 (1C), 110.0 (1C), 109.4 (1C), 69.0 (1C), 62.4 (1C), 56.9 (2C), 26.8 (1C), 26.1 (1C), 22.95 (3C), 22.89 (3C); HRMS-ESI $[M+Na]^+$ calculated for $C_{26}H_{32}N_4O_4S_2Cl_2Na^+$ 621.1140 found 621.1147.

NMR spectra analysis

Attribution of ^{1}H and ^{13}C NMR signals for compound **2a**

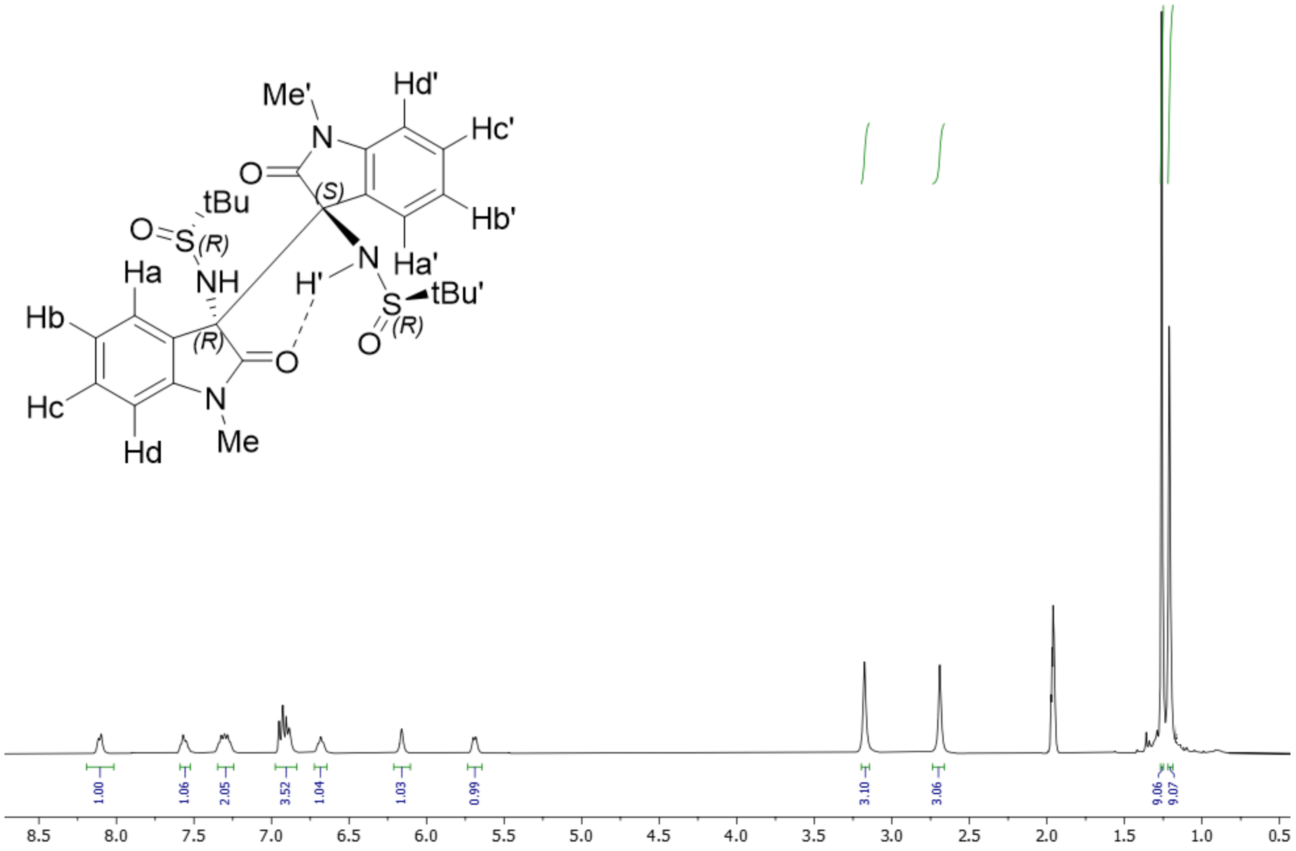

Proton	Chemical Shift *(ppm)*	Proton	Chemical Shift (ppm)
Ha	5.69	Ha’	8.11
Hb	6.68	Hb’	7.29
Hc	7.33	Hc’	7.58
Hd	6.94	Hd’	6.92
NH	6.16	NH’	6.88
Me	3.18	Me’	2.69
tBu	1.26	tBu’	1.21

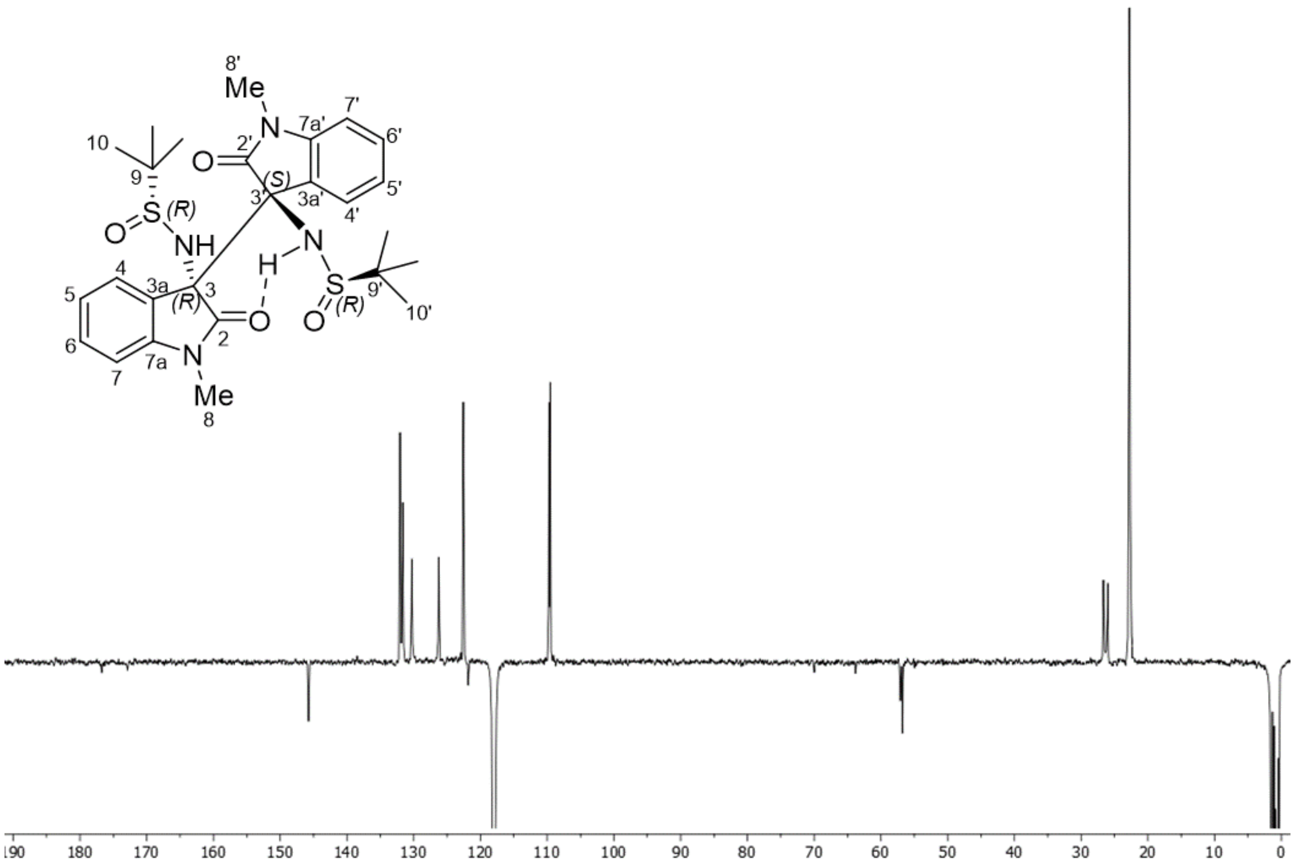

Carbon	Chemical Shift (ppm)	Carbon	Chemical Shift (ppm)
C-2	176.7	C-2’	172.9
C-3	63.8	C-3’	70.0
C-4	126.2	C-4’	130.3
C-5	122.63	C-5’	122.57
C-6	131.6	C-6’	132.1
C-7	109.7	C-7’	109.5
C-8	26.7	C-8’	26.0
C-9	57.1	C-9’	56.7
C-10	22.76	C-10’	22.79
C-3a and C-3a’	121.8	C-7a and C-7a’	145.8

Copies of ^{1}H and ^{13}C NMR spectra

^{1}H NMR spectra of compound **2a** (400 MHz, CD_3CN)

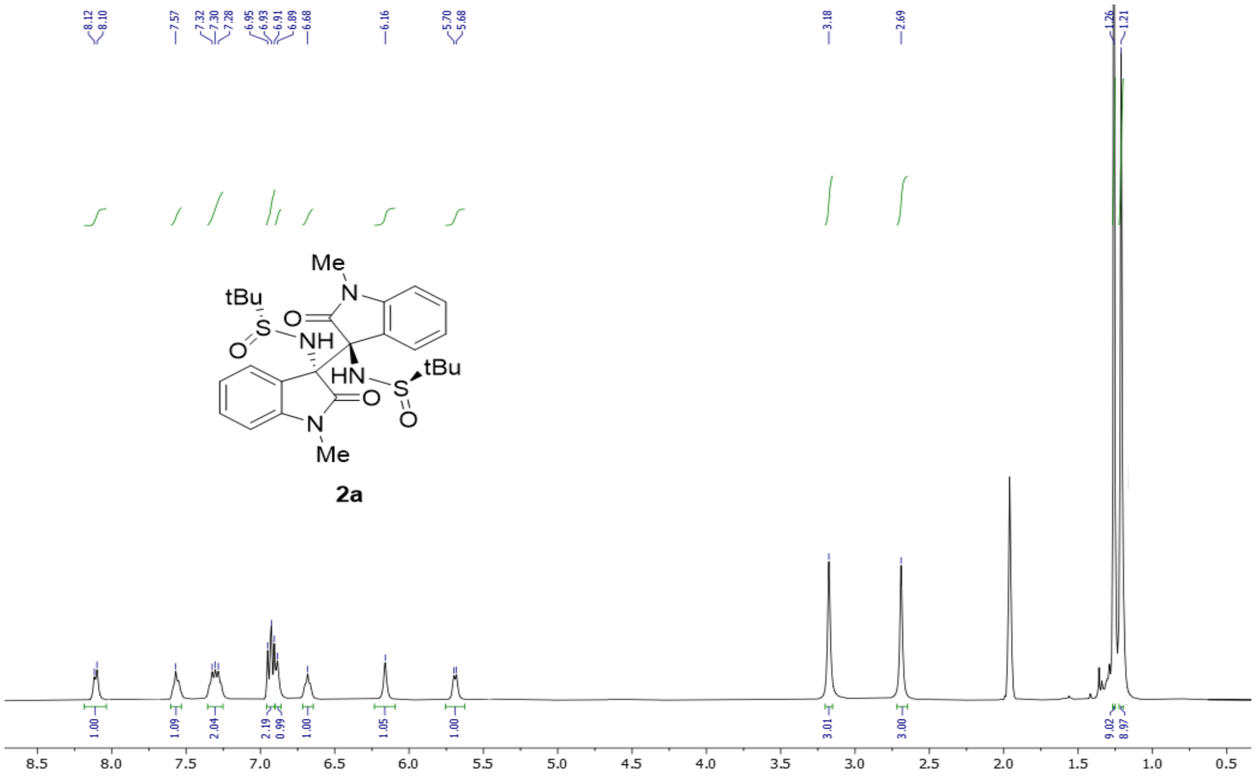

^{13}C NMR spectra of compound **2a** (101 MHz, CD_3CN)

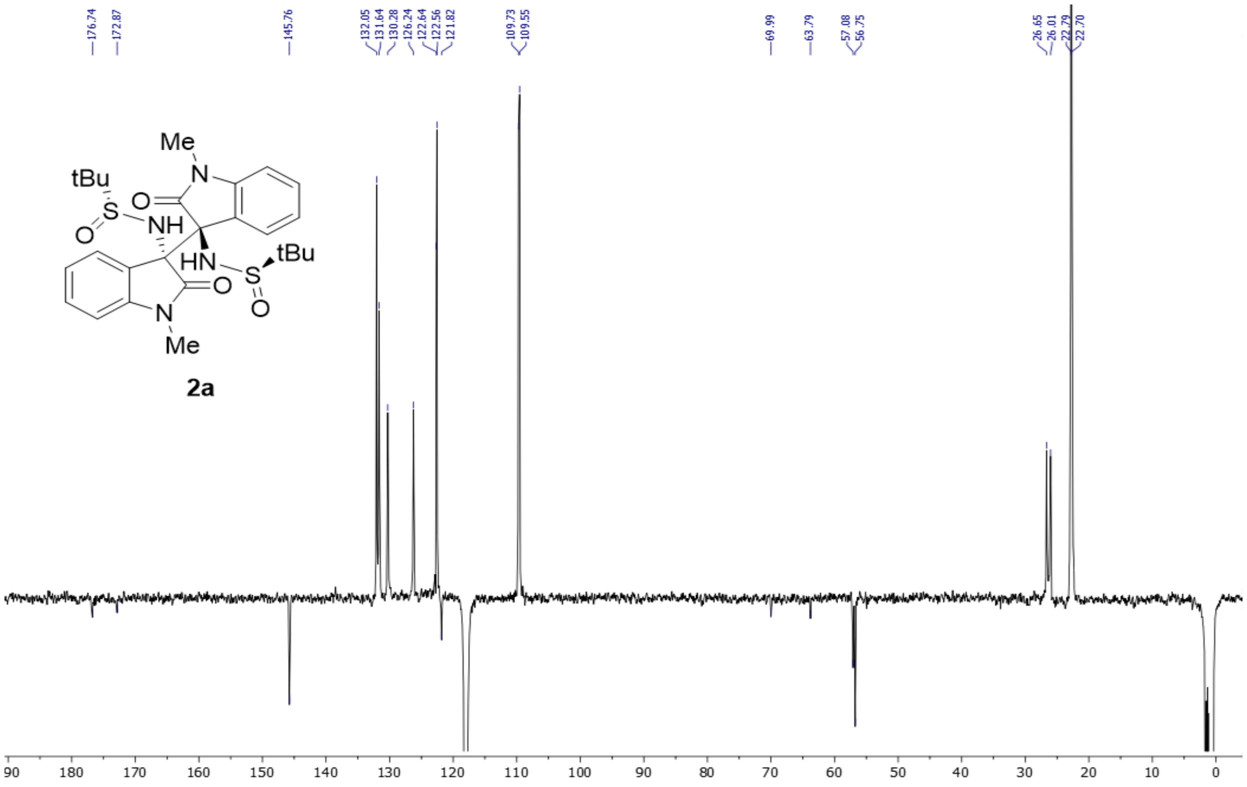

^{1}H NMR spectra of compound **2b** (400MHz, CD_3CN)

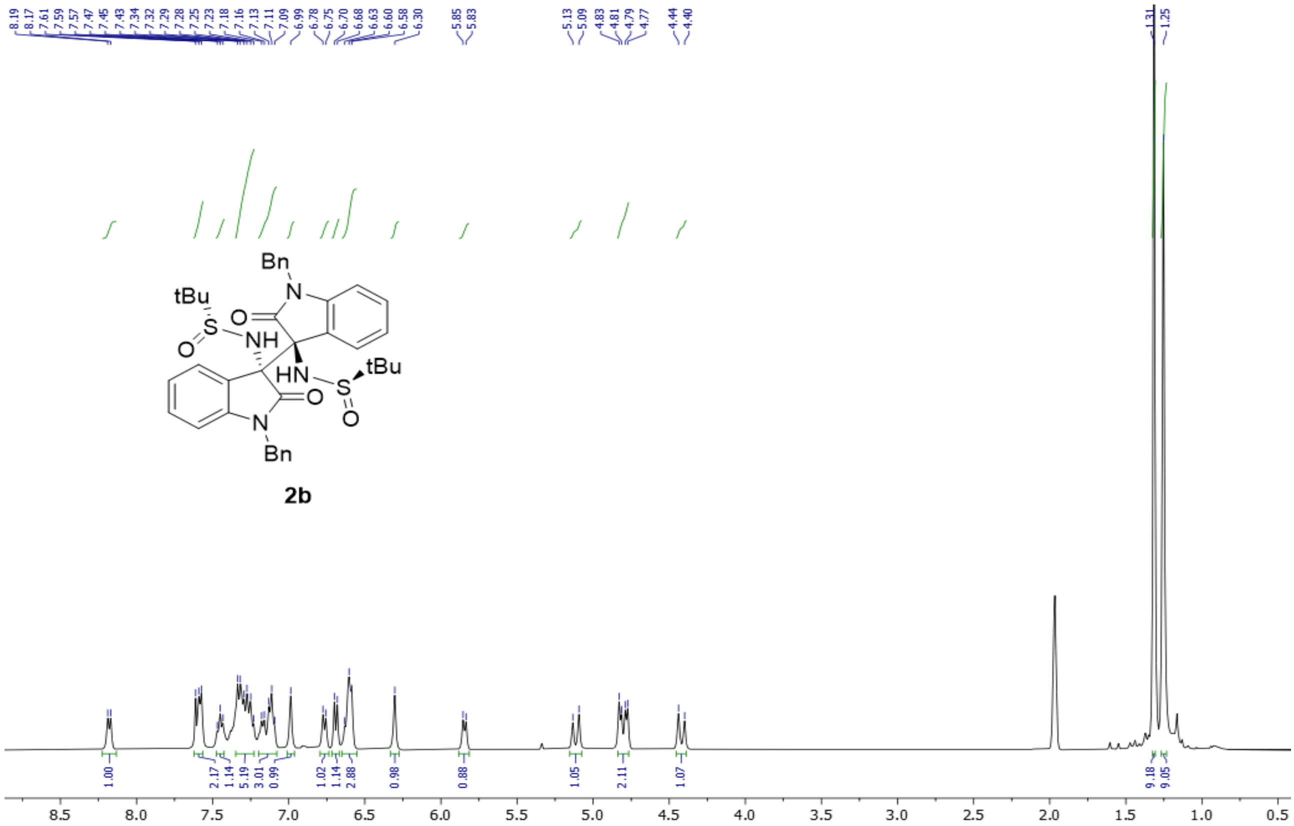

^{13}C NMR spectra of compound **2b** (101 MHz, CD_3CN)

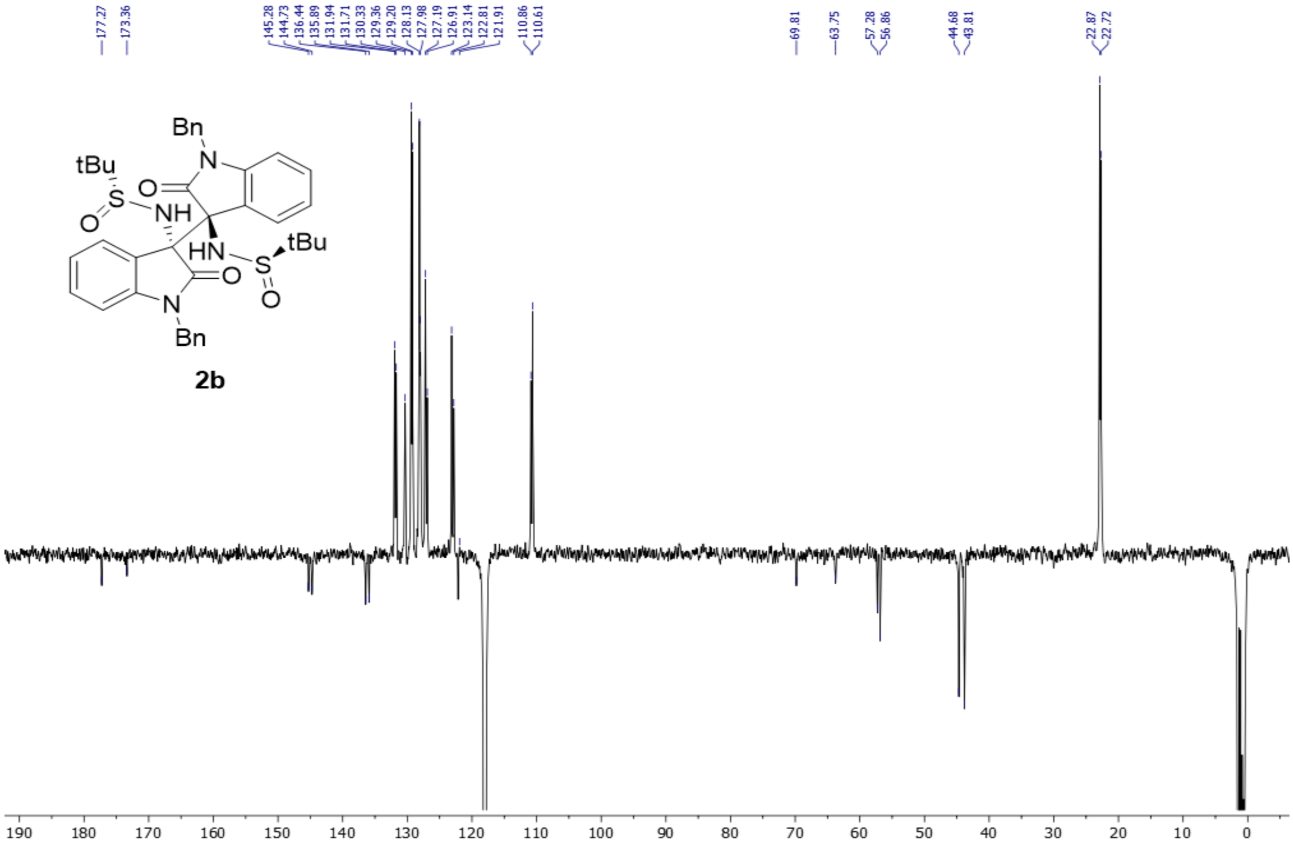

^{1}H NMR spectra of compound **2c** (400 MHz, $CDCl_3$)

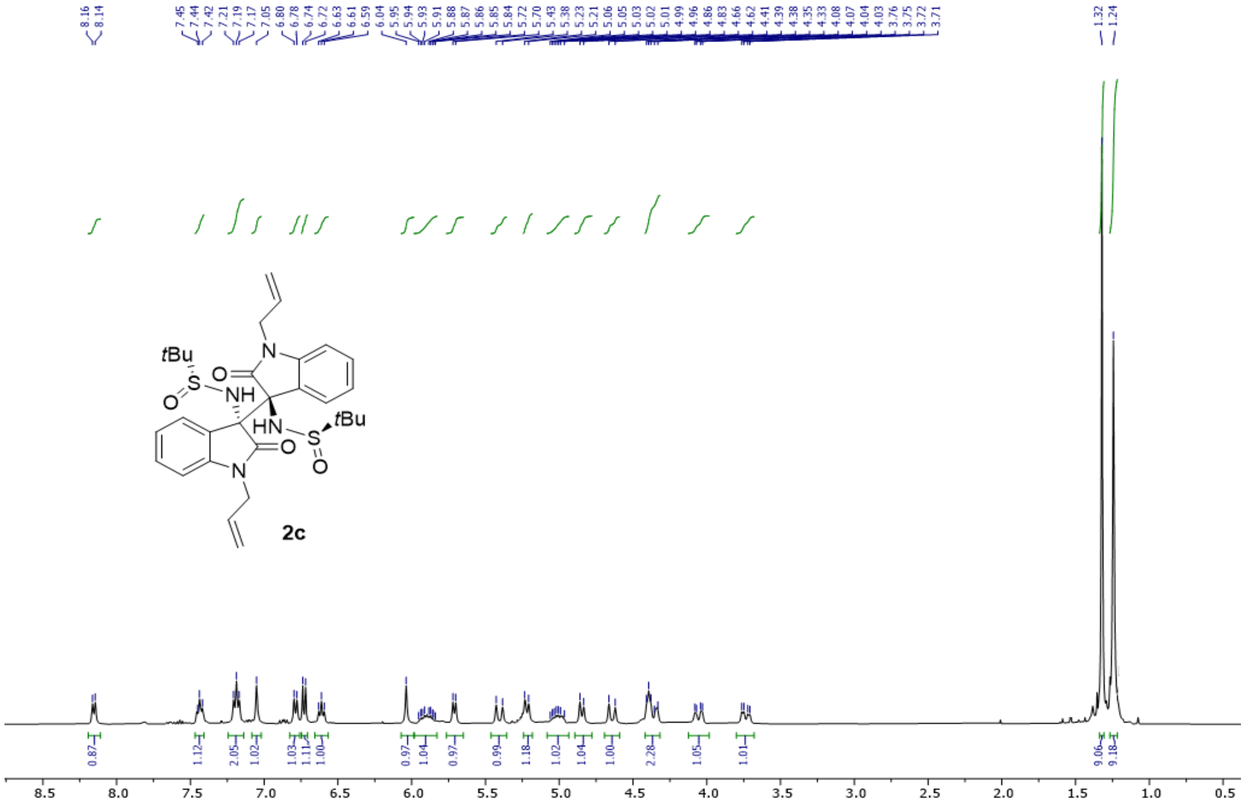

^{13}C NMR spectra of compound **2c** (101 MHz, $CDCl_3$)

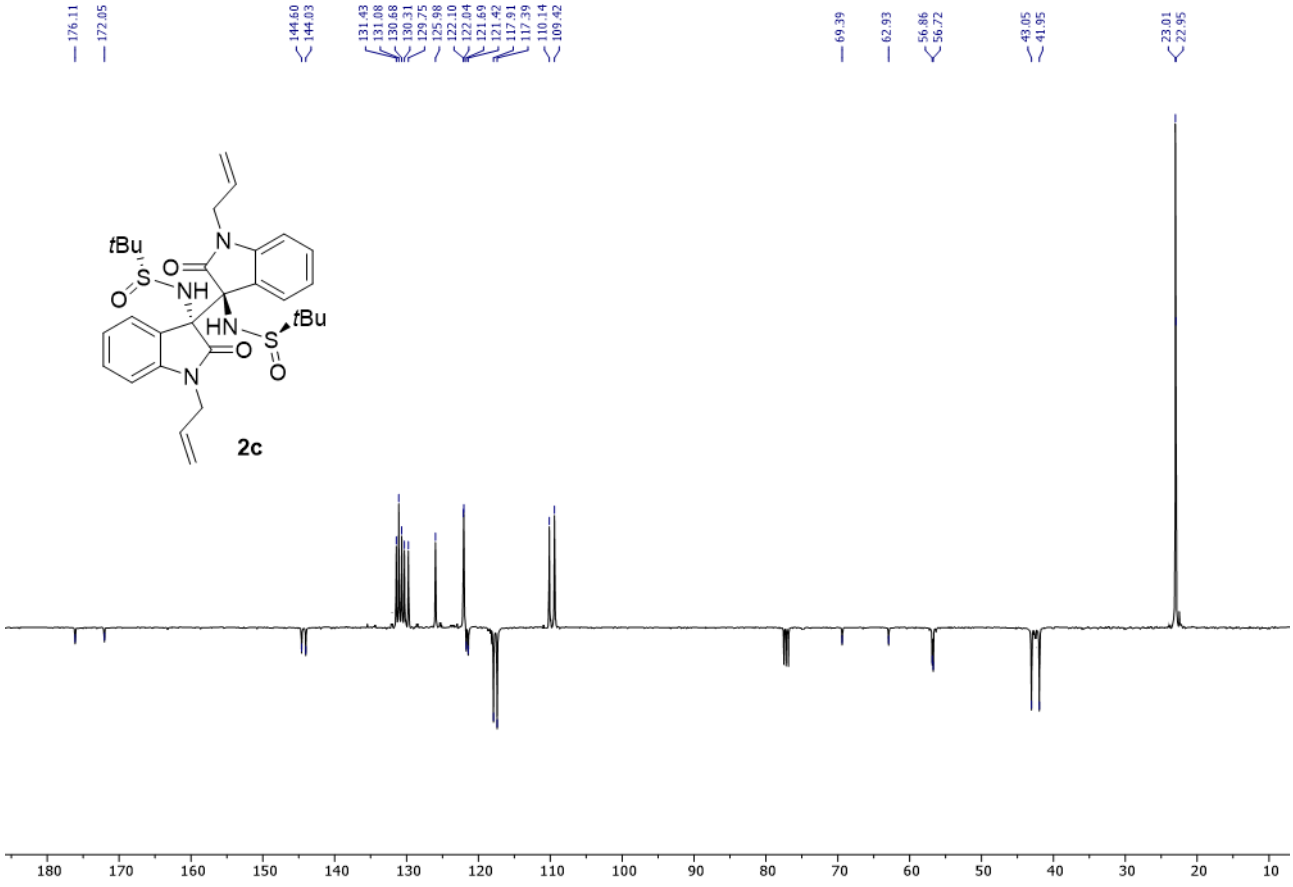

^{1}H NMR spectra of compound **2d** (400 MHz, $CDCl_3$)

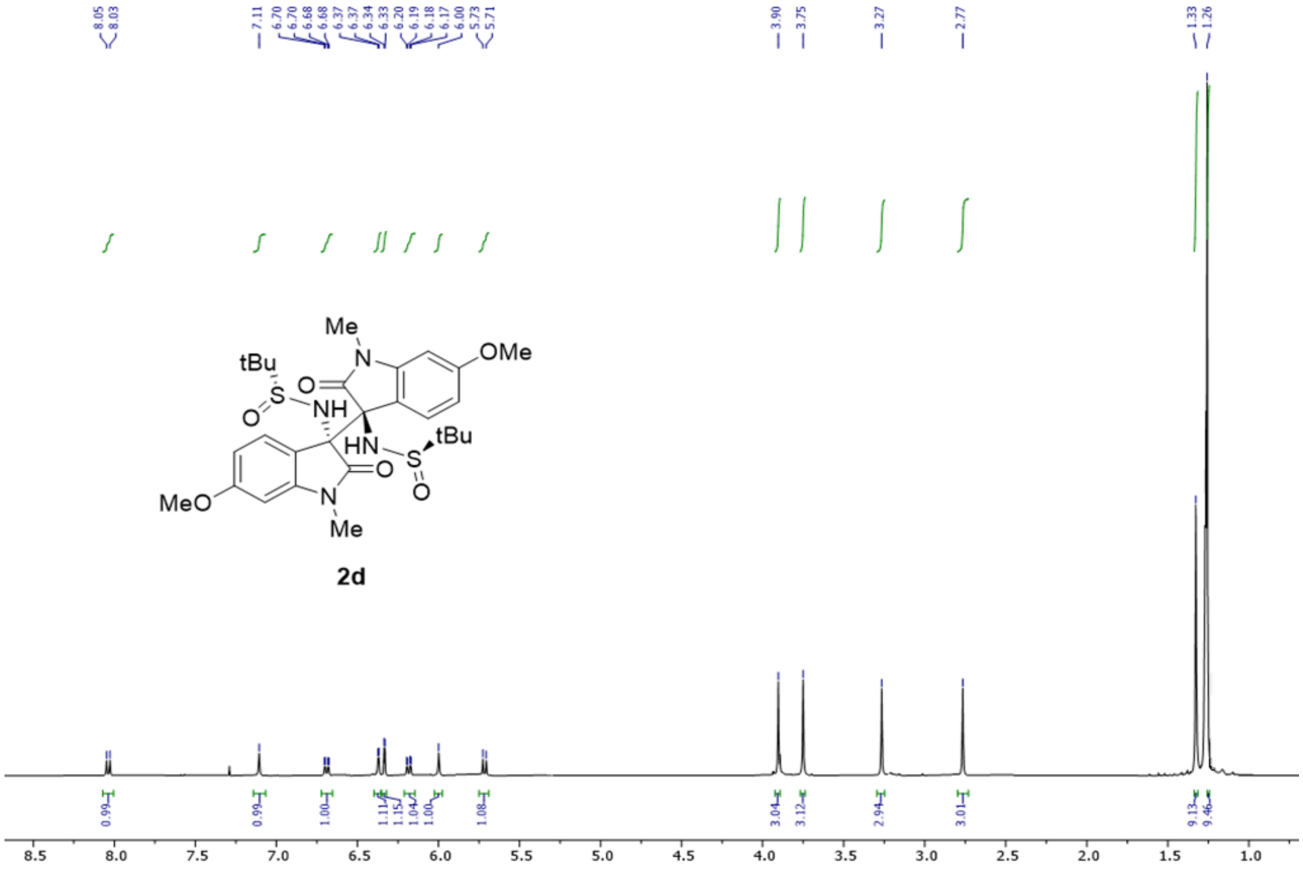

^{13}C NMR spectra of compound **2d** (101 MHz, $CDCl_3$)

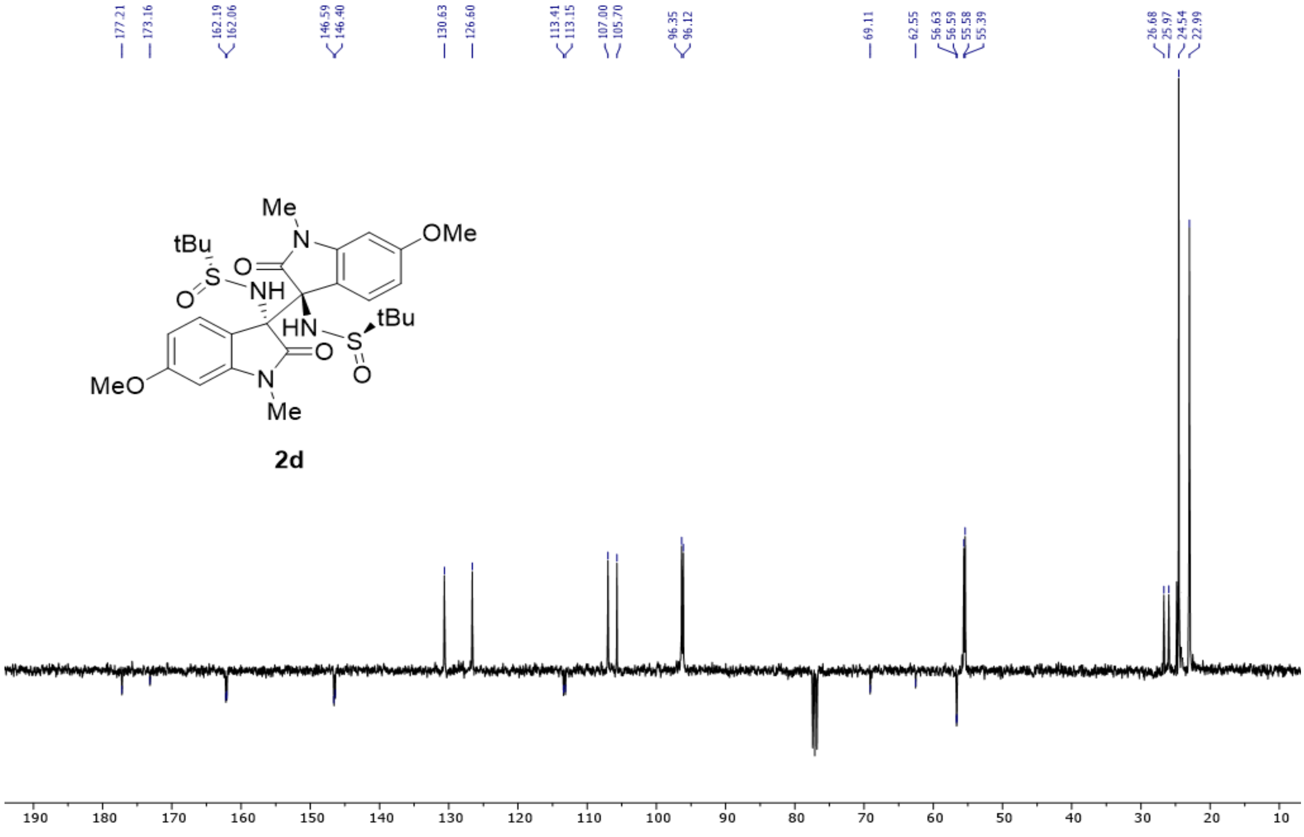

^{1}H NMR spectra of compound **2e** (400 MHz, $CDCl_3$)

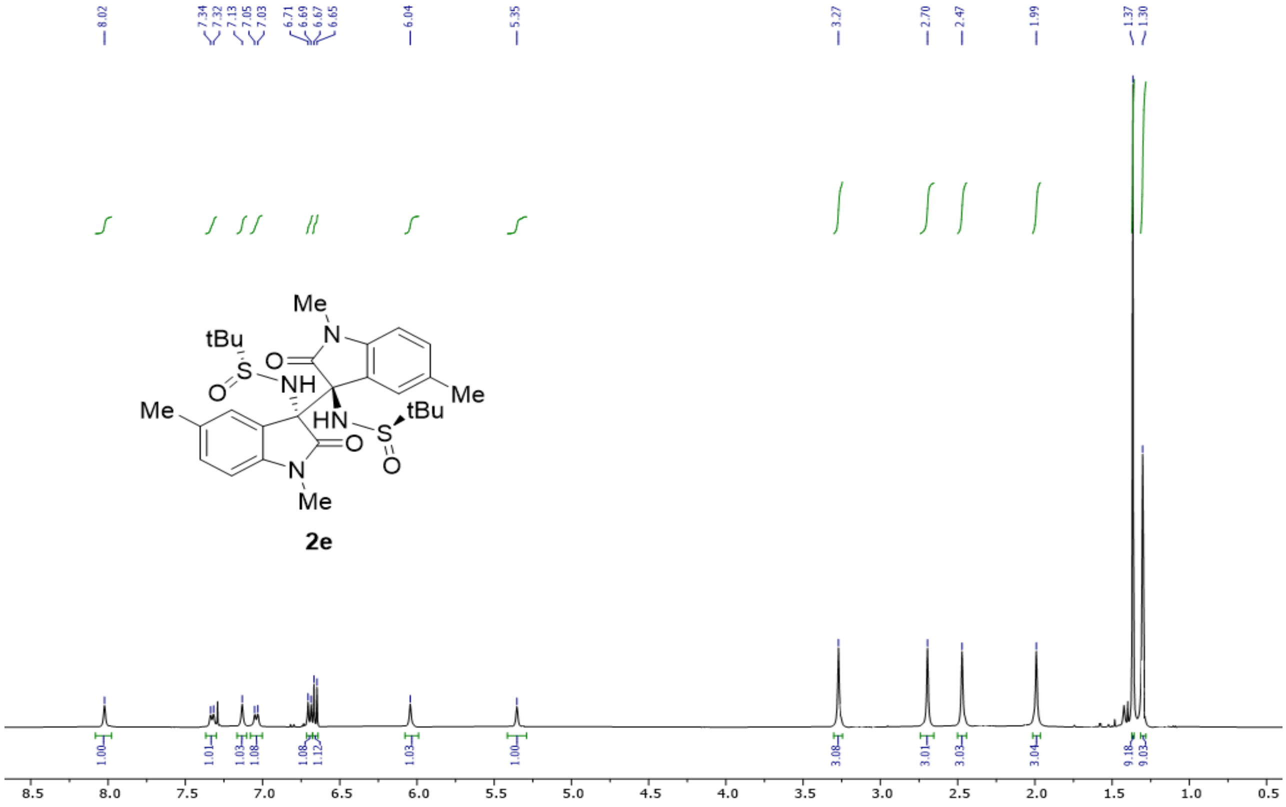

^{13}C NMR spectra of compound **2e** (101 MHz, $CDCl_3$)

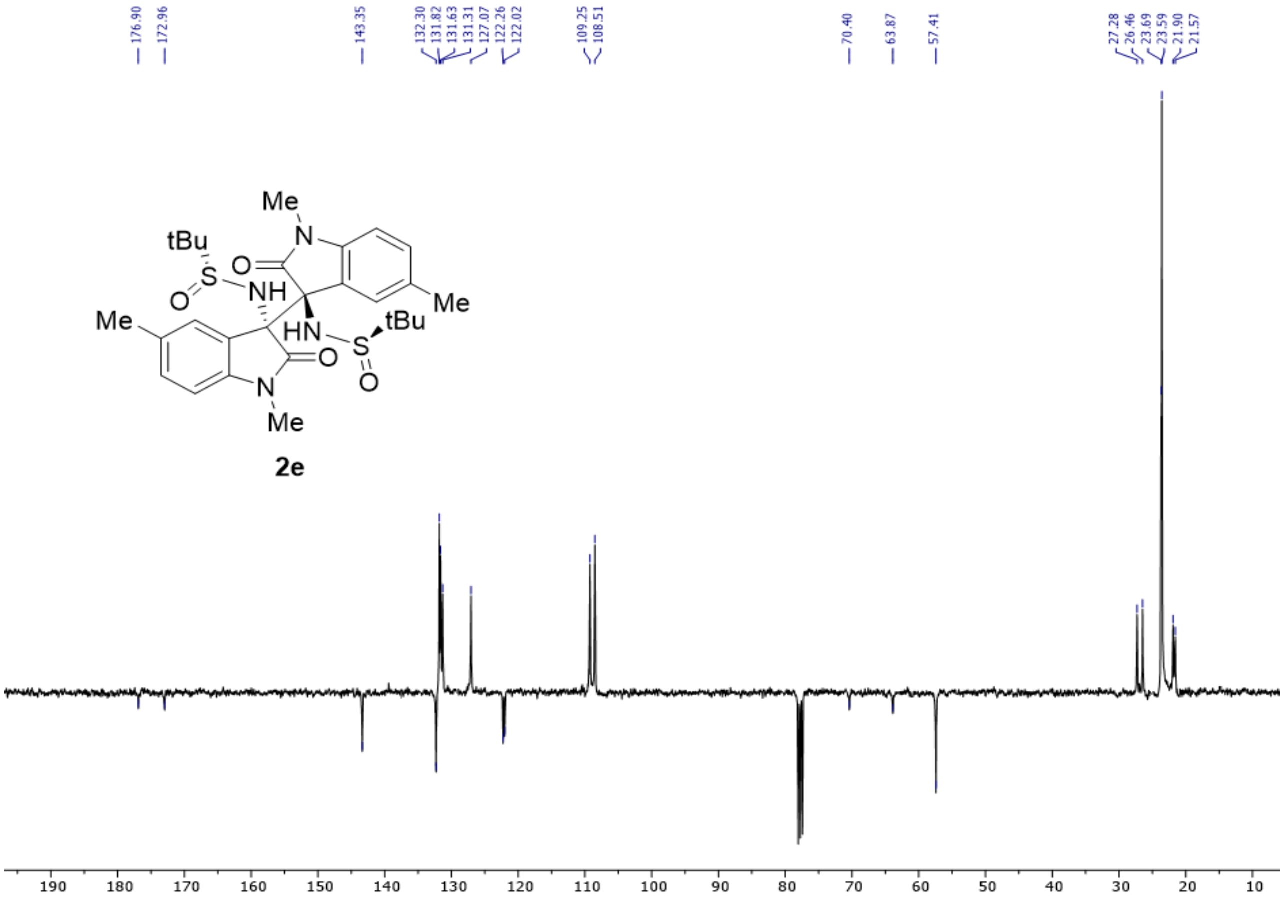

^{1}H NMR spectra of compound **2f** (400 MHz, $CDCl_3$)

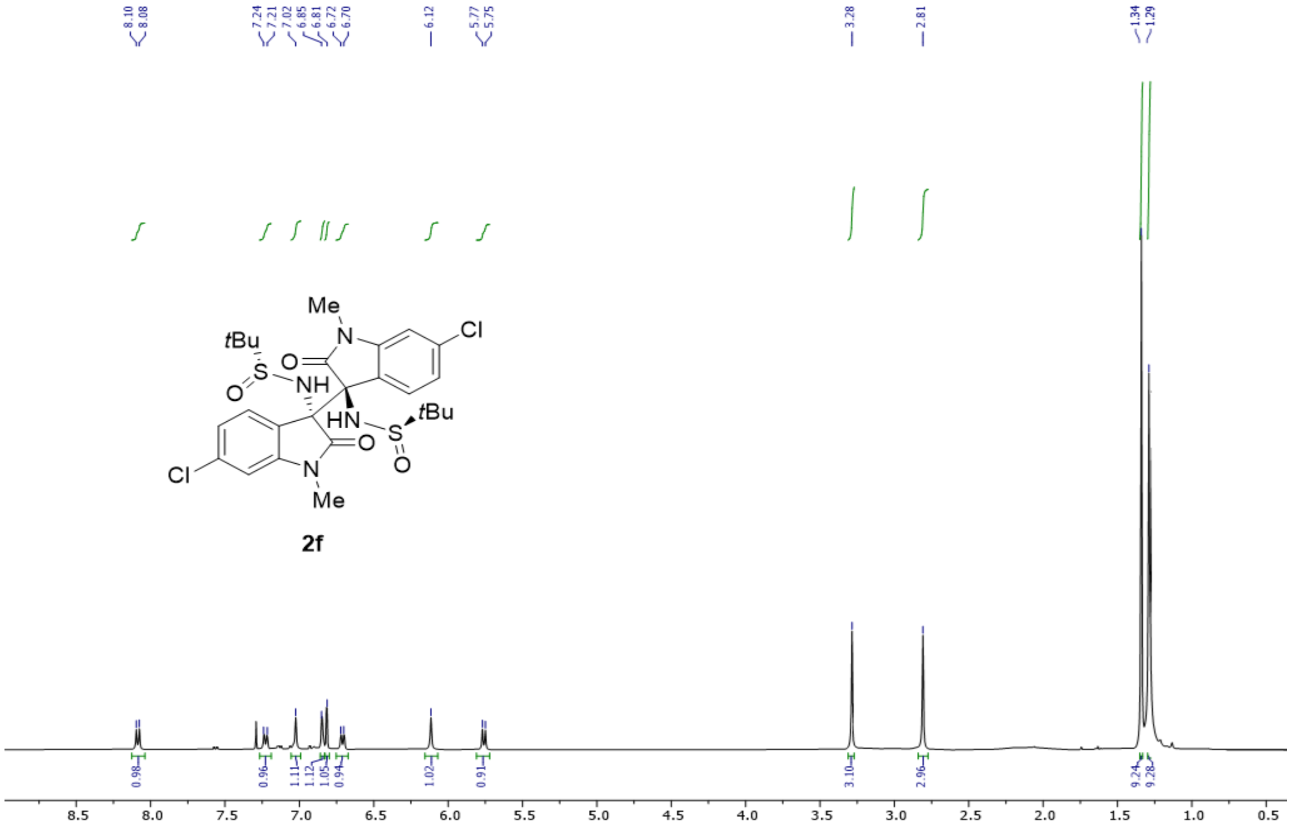

^{13}C NMR spectra of compound **2f** (101 MHz, $CDCl_3$)

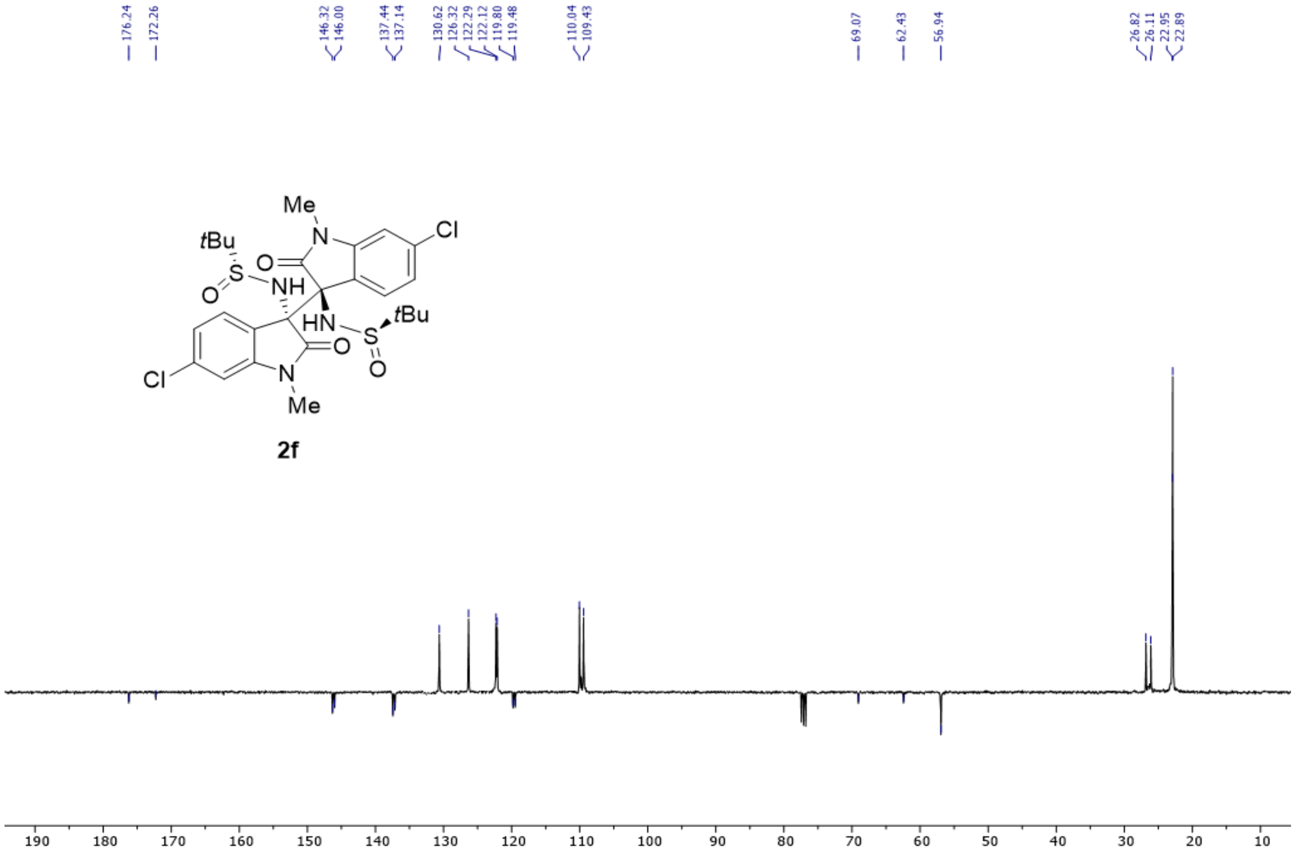

Crystallographic data for compound 2a

The sample selected for the X-ray analysis was a yellowish, transparent prism with dimensions ≈ 0.500 × 0.350 × 0.175 mm (Figure S2). It was mounted on a glass fibre, using perfluorinated oil as a glue.

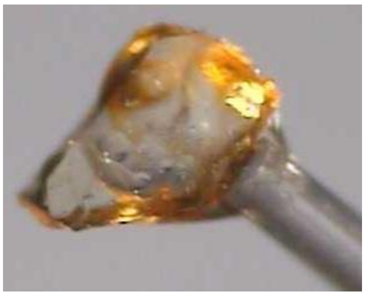

Figure S2. Specimen employed in the X-ray experiment. Real dimensions: 0.500 × 0.350 × 0.175 mm

The data collection was carried out with a Bruker AXS Smart Apex three-circle diffractometer, equipped with a CCD area detector. Graphite-monochromated Mo K_α radiation (λ=0.71073 Å) was employed at a nominal power of 50 kV × 30 mA of the X-ray source. The data collection consisted in 5 redundant ω-scans at steps of 0.25 deg for an exposure time of either 10 s/frame or 20 s/frame, all performed in ambient conditions. Eventually, we explored a 98.8 % complete full sphere of data, up to a maximum $\sin\vartheta/\lambda$ of 0.72 $Å^{-1}$. The raw dataset consisted of 38838 measured reflections, 91 of which were rejected as systematic absence violations. A negligible twin component was present, which was ignored in the subsequent data reduction. The data were integrated with the commercial SAINT+ program[4] and corrected for absorption (μ = 0.230 mm^{-1}) and anisotropic beam intensity with SADABS.[5] The final dataset consisted of 8556 independent reflections (5979 with $I > 2\sigma(I)$) with R_{int} = 0.0373. The space group was unequivocally established by systematic absence relationships.

The substance **2a** crystallizes in $P2_12_12_1$ (system orthorhombic), with unit cell parameters a = 10.826(2) Å, b = 13.862(2) Å, c = 18.387(3) Å, V = 2759(1) $Å^3$, density 1.277 g/cm^3. The structure was solved by direct methods by Shelxs and refined by least squares within the independent atom model approximation implemented in Shelxl[6]. The final crystallographic agreement factors were Goodness-of-fit: 1.085, R1(F) = 0.0547 for 5979 $F_o > 4\sigma(F_o)$, and R1(F) = 0.0802, wR(F^2)= 0.1634 for all the 8556 independent data. Maximum and minimum Fourier residuals read as $\Delta\rho_{MAX/MIN}$ = +0.20/ –0.55 e/$Å^3$.

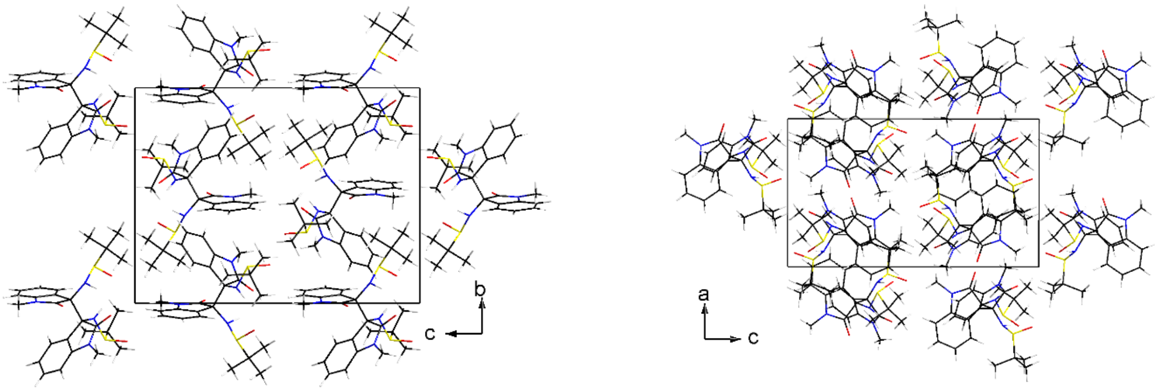

(a) (b)

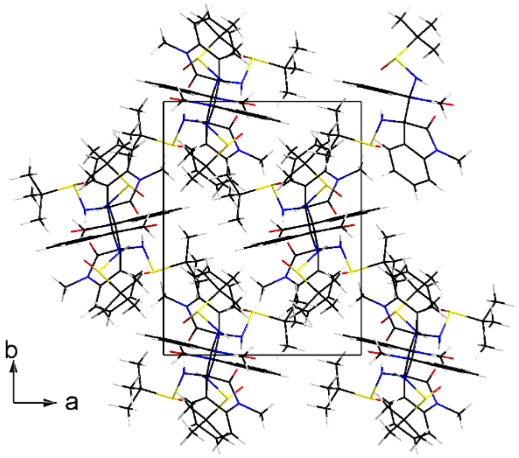

(c)

Figure S3. Wire–stick representation of the crystal packing of **2a** at rt, as seen along the *a* (a), *b* (b) and *c* (c) cell axes. Colour code as in Figure 1 (main text).

Discussion

The compound is chiral and crystallizes in the orthorhombic Sohncke space group $P2_12_12_1$ with 4 formulae in cell and 1 molecule in the asymmetric unit. The absolute configuration of the two sulfur stereogenic centres is confirmed to be R (see main text). The Flack parameter, computed by classical fits against all the intensities[7,8], refines to 0.1(1).

The C2–C10 single bond (numbering scheme in Figure 1, main text) is quite long (1.5863(2) Å), as expected due to crowding of the two facing oxindole systems. In the crystal, the mean least-squares planes across the C–N backbones of the two indole rings are mutually rotated by ≈48.6 deg. At the same time, NH groups set up intramolecular hydrogen bonds with the O acceptors of the sulfinamide moieties (see main text). Likely, such hydrogen bonds are crucial to stabilize the conformer despite a minor intramolecular H3N···H21C steric clash of the N3 amine with the C21 methyl ($d_{H\cdots H}$ = 1.89 Å).

Figure 2 shows the main packing motifs in the (*b,c*), (*a*,*c*) and (*a*,*b*) planes. The only relevant hydrogen bond donors are the NH moieties in the sulfinamide groups, while both S=O and the keto group in the 2-oxindole rings can serve as acceptors. However, both donors are saturated by intramolecular hydrogen bonded contacts (Table 1, main text), resulting in no extended HB patterns through the crystal. Also, no relevant stacking interactions can be found, due to the misalignment of the neighbouring oxindole systems (see above). Consequently, the main structure-determining interactions are expected to be van der Waals and electrostatic interactions.

References

[1] S. Elderwish, A. Audebrand, C.G. Nebigil and L. Dèsaubry. Discovery of 3,3’-pyrrolidinyl-spirooxindoles as cardioprotectant prohibitin ligands. (2020) *Eur. J. Med. Chem. 186,* 111859.
[2] W. Yan, D. Wang, J. Feng, P. Li, R. Wang. Zinc-mediated diastereoselective synthesis of 3-amino oxindoles by addition of methyl and terminal alkynes to N-tert-butanesulfinyl ketimines. (2012) *J. Org. Chem. 77*, 3311.
[3] G. Rainoldi, M. Faltracco, C. Spatti, A. Silvani and G. Lesma. Organocatalytic access to enantioenriched spirooxindole-based 4-methyleneazetidines. (2017) *Molecules 22*, 2016/1-2016/13.
[4] Bruker (2012). *SAINT+*. Bruker AXS Inc., Madison, Wisconsin, USA.
[5] Bruker (2018). *SADABS.* Bruker AXS Inc., Madison, Wisconsin, USA.
[6] Sheldrick, G. M. (2015). *Acta Cryst.* C**71**, 3-8.
[7] Flack, H. D. & Bernardinelli, G. (1999). Acta Cryst. A55, 908-915
[8] Flack, H. D. & Bernardinelli, G. (2000). J. Appl. Cryst. 33, 1143-1148
